# GIMAP5 Deficiency Is Associated with Increased AKT Activity in T Lymphocytes

**DOI:** 10.1371/journal.pone.0139019

**Published:** 2015-10-06

**Authors:** Xi-Lin Chen, Daniel Serrano, Marian Mayhue, Kasper Hoebe, Subburaj Ilangumaran, Sheela Ramanathan

**Affiliations:** 1 Immunology Division, Department of Pediatrics, Faculty of Medicine and Health Sciences, Université de Sherbrooke, Sherbrooke, J1H 5N4, Québec, Canada; 2 Department of Pediatrics, Division of Cellular and Molecular Immunology, Cincinnati Children's Hospital Medical Center, Cincinnati, OH 45229, United States of America; 3 Centre de Recherche Clinique, Université de Sherbrooke, Sherbrooke, J1H 5N4, Québec, Canada; University of Iowa, UNITED STATES

## Abstract

Long-term survival of T lymphocytes in quiescent state is essential to maintain their cell numbers in secondary lymphoid organs. In mice and in rats, the loss of functional GTPase of the immune associated nucleotide binding protein 5 (GIMAP5) causes peripheral T lymphopenia due to spontaneous death of T cells. The underlying mechanism responsible for the disruption of quiescence in *Gimap5* deficient T cells remains largely unknown. In this study, we show that loss of functional *Gimap5* results in increased basal activation of mammalian target of rapamycin (mTOR), independent of protein phosphatase 2A (PP2A) or AMP-activated protein kinase (AMPK). Our results suggest that the constitutive activation of the phosphoinositide 3-kinase (PI3K) pathway may be one of the consequences of the absence of functional GIMAP5.

## Introduction

The GTPase of immune-associated protein (*Gimap*) ^||^ family is predominantly expressed in lymphocytes and cells of the hematopoietic system [1,2]. In diabetes prone Bio-Breeding (BB-DP) rats, the *lyp* allele arises from a frame-shift mutation within the *Gimap5* gene that deletes 223 amino acids at the C-terminus [3,4]. The life span of T cells is reduced in the periphery of *Gimap5* ^*lyp/lyp*^ rats resulting in a profound T lymphopenia in the secondary lymphoid organs [5–7]. Two independently generated lines of *Gimap5* deficient mice also exhibit progressive loss of T cell populations [8,9]. Whereas the cell survival defect is confined to T cells in *Gimap5* ^*lyp/lyp*^ rats, mice lacking *Gimap5* show defects in various hematopoietic cell types including a breakdown of quiescence in hematopoietic stem cells [8–10]. Despite a decade of efforts by several groups, mechanisms underlying the pro-survival function of GIMAP5 remain unclear.

Different pathways that contribute to the maintenance of quiescence dictate the lifespan of naïve T cells in the periphery. Basal homeostatic signals through the T cell receptor (TCR) and interleukin–7 receptor (IL-7R) are required to maintain the survival of post-thymic naive T lymphocytes [11–15]. IL–7 promotes T cell survival through multiple downstream signaling pathways including Janus kinase/signal transducers and activators of transcription (JAK/STAT) pathway and PI3K/AKT pathway by increasing the expression of anti-apoptotic proteins such as BCL–2 and MCL1 [16]. The TCR-dependent survival signals remain less clear although they are known to require LCK, a non-receptor tyrosine kinase that is activated following TCR stimulation by foreign antigens [14]. Similarly absence of KLF2 and certain other genes also compromises survival of naïve T cells [17].

In addition to T cell-specific molecules, classical pathways involving liver kinase B1 (LKB1) and AMPK, that mediate survival in most of the cell types, are also required for the survival of T cells [18–20]. The quiescent state that promotes naïve T cell survival is accompanied by a catabolic metabolism and low mTOR activity [21,22]. LKB1 and AMPK regulate cellular energy metabolism and cell polarity by activating tuberous sclerosis complex 1/2 (TSC1/2) that suppresses mTOR complex 1 (mTORC1) [20,23,24]. In contrast, activation of AKT, following engagement of the TCR complex at the immunological synapse, phosphorylates the TSC1/2 complex, thereby releasing small GTPase RAS homologue enriched in brain (RHEB) from suppression to activate the mTORC1 [25]. Activated mTORC1 promotes translation and protein synthesis by activating 70-kDa ribosomal S6 kinase (S6K1) and releasing the suppression of eukaryotic initiation factor 4E (eIF-4E) by the repressor protein eIF-4E binding protein 1 (4EBP1) [26]. Several studies have shown that deficiency of LKB1 or TSC1/2 leads to high mTORC1 activity and loss of T cell quiescence [18,23,24,27,28]. While the pathways leading to the activation of the mTORC1 complex following engagement of the TCR at the immunological synapse is well-characterized, it is not clear how homeostatic signals through the IL-7R and TCR molecules are integrated in T cells to promote quiescence and survival.

Our previous observations suggest that GIMAP5-deficient T cells may be ineffective in integrating homeostatic signals through the TCR complex [29,30]. Even though the pattern of tyrosine phosphorylation following cross-linking of CD3/CD28 complex was comparable between T cells from control and *Gimap5* ^*lyp/lyp*^ rats, T cells from the mutant rats showed reduced calcium (Ca^2+^) influx from the extracellular medium. This decrease was associated with a reduction in the ability of the mitochondria to buffer the cytosolic Ca^2+^ [30]. While *Gimap5* mutation does not affect the proliferation of T cells in the rats, in mice, the proliferative response is severely decreased following activation through the TCR/CD3 complex [8,9]. T cells from *Gimap5* ^*sph/sph*^ mice exhibit progressive loss of forkhead box O (FOXO) proteins with age [31]. While analyzing the signaling pathways that are activated following TCR stimulation in T cells from *Gimap5* mutant rats and mice, [32–34] we detected phosphorylated AKT even in the absence of any stimulation. Here we report that *Gimap5* deficiency results in the constitutive activation of the AKT/mTORC1 pathway.

## Materials and Methods

### Animals

*Gimap5* ^*sph/sph*^ mice [9] were crossed with OT-II TCR transgenic mice to generate OTII *Gimap5* ^*sph/sph*^ mice. As *Gimap5* ^*sph/sph*^ mice do not survive beyond 3–4 months, female *Gimap5* ^*sph/+*^ mice were crossed with male *Gimap5* ^*sph/+*^ mice. C57Bl/6 mice were purchased from Charles River Canada. *Gimap5* ^*+/+*^ and *Gimap5* ^*lyp/lyp*^ rats in the ACI.1u background have been described before [35]. Mice and rats were bred and maintained under specific pathogen free conditions at the Université de Sherbrooke. The institutional animal ethics committee approved all the protocols (protocol number 050-13B).

### Reagents

LY294002 (LY), oligomycin, fluoro-carbonyl cyanide phenylhydrazone (FCCP), rotenone, antimycin A, RPMI–1640 cell culture medium, fetal bovine serum (FBS) and antibody against actin were from Sigma Aldrich. Thapsigargin (TG), rapamycin, okadaic acid (OA), CAL–101 and calyculin A were from Calbiochem. OVA_323-339_ peptide (ISQAVHAAHAEINEAGR) was custom synthesized by Genscript (New Jersey, USA). ATP detection kit, Dynabeads CD4 T cell kit for negative selection and dynabeads pan mouse IgG were from Life Technologies. Anti-mouse CD3 (2C11), anti-mouse CD28 (37.51), anti-rat TCR (R73) and anti-Rat CD28 (JJ319) were obtained from BD Pharmingen Biosciences. Goat anti-hamster and goat anti-mouse antibodies for cross-linking were purchased from Jackson ImmunoResearch Laboratories. PP2A immuno-precipitation phosphatase assay kit was from Upstate/Millipore. Enhanced chemiluminescence (ECL) was obtained from GE Healthcare. Antibodies to phosphorylated and total AMPK**α**(Thr172), AMPKβ1(Ser108), Acetyl CoA carboxylase(ACC) (Ser79), S6(S235/236), 4EBP1(Thr37/46), AKT(S473) and FOXO1(T24) were obtained from Cell Signaling Technology.

### T cell preparation and stimulation

CD4^+^ T cells were used for all the experiments as the paucity of CD8^+^ T cells in *Gimap5* mutant rats and mice precludes their isolation in sufficient numbers to carry out any experiment. Single-cell suspension from pooled lymph nodes (inguinal, axillary, brachial, mesenteric and cervical) of mice or rats were suspended in PBS supplemented with 2% FCS. CD4^+^ T cells were purified by negative selection using magnetic beads following manufacturer’s instructions (Dynabeads, Life Technologies). T cells were stimulated by crosslinking with the anti-CD3 and or anti-CD28 mAb as required. Goat anti-hamster or anti-mouse antibodies were used as cross-linking agents for T cells from mice and rats, respectively. OT-II CD4^+^ T cells were stimulated with OVA323-339 peptide (0.5μM) presented by irradiated splenocytes that served as antigen-presenting cells (APC) for the indicated duration. In experiments using inhibitors, cells were pre-incubated in the presence or absence of the inhibitors in complete RPMI containing calcium for 30 min at 37°C. The vehicle control was PBS or DMSO that was diluted to the same concentration. The concentration of DMSO did not exceed 0.1%. Purified CD4^+^ T cells obtained from a single *Gimap5* mutant rat or mouse are not sufficient to carry out analyses of signaling pathways. Therefore, lymph nodes were pooled from two *Gimap5* ^*sph/sph*^ mice for every *Gimap5* ^*+/+*^ mouse and from three *Gimap5* ^*lyp/lyp*^ rats for every control rat in any given experiment. Pooled samples were prepared for every repeat experiment. In all experiments, mice and rats were used at 4 weeks of age. Animals were sex-matched within a given experiment, even though both males and females were used.

### PP2A activity assay

The PP2A protein phosphatase activity of CD4^+^ T cellular lysates was determined by measuring the generation of free phosphate from the threonine phospho-peptide using PP2A immunoprecipitation phosphatase assay kit. Cell lysates were prepared in lysis buffer (20 mM imidazole-HCL, 2 mM EDTA, 2 mM EGTA, PH7.0 with protease inhibitor cocktail). Five hundred micrograms of total cell lysate was immunoprecipitated with anti-PP2A antibody. PP2A activity in the immunoprecipitates was determined using 750 μM phospho-peptide as substrate. After 10 minutes of incubation at 30°C malachite dye was added, and free phosphate was measured by the relative absorbance at 650 nM.

### Measurement of Oxygen consumption rates (OCR)

CD4^+^ T cells were stimulated overnight with plate-bound anti-CD3/CD28 and oxygen consumption rate (OCR) (in pmol/min) was measured using Seahorse XF–96 metabolic extracellular flux analyzer (Seahorse Bioscience). Briefly, CD4^+^ T cells were re-suspended in XF assay buffer and were plated onto Seahorse cell plates (6 × 10^5^ cells per well). T cell attachment was enhanced by centrifugation. Cells were incubated at 37°C in a CO_2_-free incubator for one hour prior to measurements. Then OCR was measured under basal conditions and following addition of oligomycin (1 μM), FCCP (1.5 μM) and rotenone (100 nM) + antimycin A (1 μM).

### ATP assay

The cellular levels of ATP were measured using a bioluminescence assay kit according to the manufacturer's instructions. Briefly, rat CD4^+^ T cells were untreated or stimulated with antibodies to CD3 and CD28 for the indicated times. The cells were collected and lysed in lysis buffer. The lysates were centrifuged at 13,000×*g* for 15 min at 4°C and supernatants were collected. ATP concentration was measured in 0.5 μg protein lysate. The luminescence was measured in a Luminometer by the absorbance at 560nM.

### SDS-PAGE and Western blot

Cells were washed with cold PBS, harvested and re-suspended in SDS-PAGE sample buffer (50 mM Tris pH 6.8, 1% (w/v) SDS, 1 mM EDTA, 1 mM dithiothreitol). Equivalent amounts of proteins were separated in SDS-PAGE gels and transferred to polyvinylidene difluoride membranes. The blots were probed with primary antibodies followed by incubation with the appropriate HRP-conjugated secondary antibodies and developed with ECL reagent. After incubating in stripping solution (2% SDS, 62.5 mM Tris pH 6.8, 100 mM 2-mercaptoethanol) for 30 min at 55°C, the blots were blocked and re-probed for total proteins.

### Statistical analysis

Data were analyzed using two-way ANOVA for statistical comparisons. p values less than 0.05 were considered statistically significant.

## Results

### *Gimap5*-deficient T cells show constitutive mTORC1 activity

**Fig 1:**
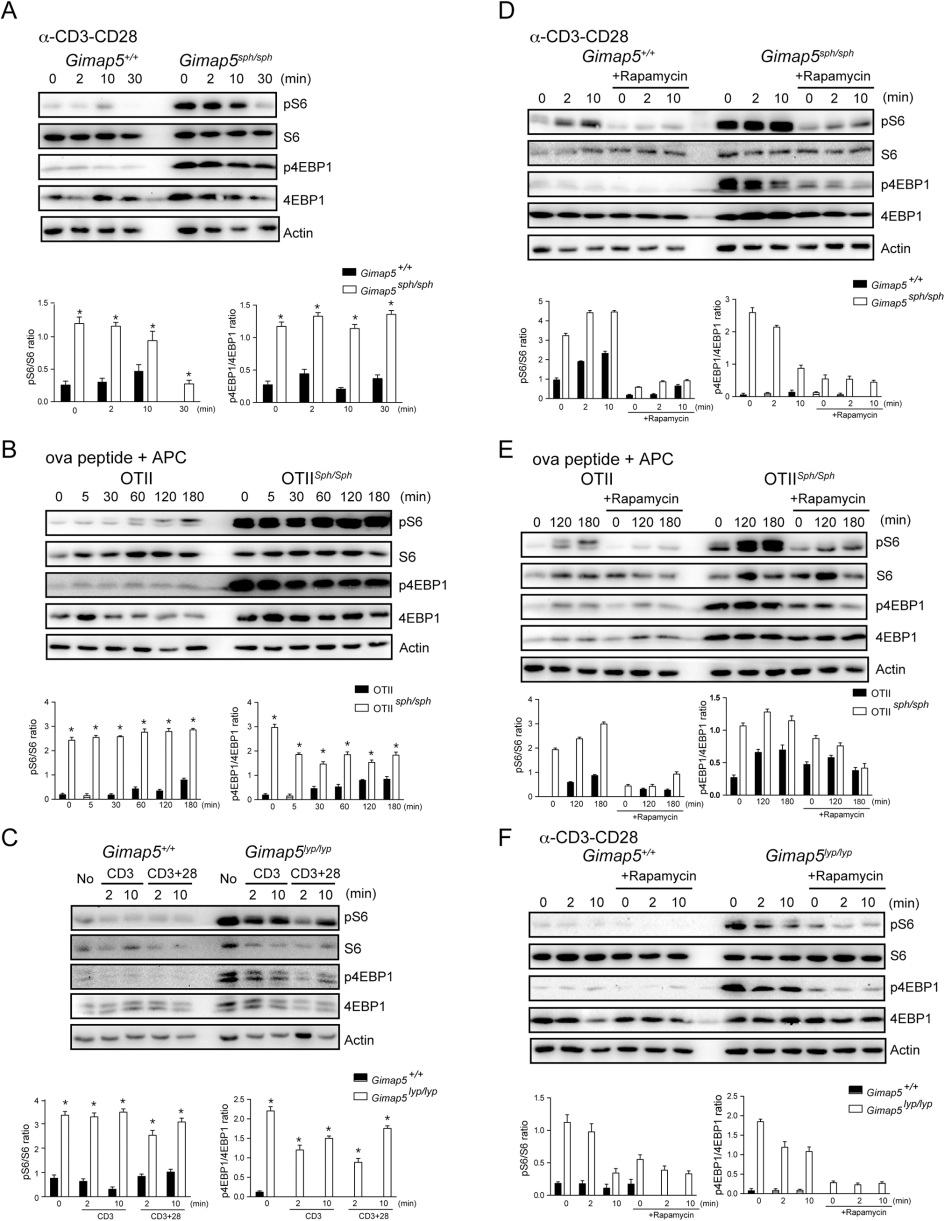
GIMAP5 regulates the activity of mTORC1. (A and C) CD4^+^ T cells from control and *Gimap5* deficient mice (A) and rats (C) were left un-stimulated or stimulated with 5 μg/μL anti-CD3 or anti-CD3/CD28 for the indicated period. Phosphorylation and the total amount of the indicated proteins in the whole cell lysates were evaluated by Western blot. (B) CD4^+^ T cells from the OTII TCR-transgenic control and *Gimap5* ^*sph/sph*^ mice were co-cultured with irradiated APC pulsed with indicated period were lysed and analyzed by Western blot. (D and F) CD4^+^ T cells from control and *Gimap5* deficient mice (D) and rats (F) were treated with vehicle or 200 nM rapamycin for 30 min followed by TCR cross-linking at different time points. Cell lysates were examined by Western blot using the indicated antibodies. (E) CD4^+^ T cells from the OTII *Gimap5* ^*sph/sph*^ and control mice were pretreated with vehicle or 200 nM rapamycin for 30 min before simulation with ova peptide for the indicated duration. Cell lysates were probed with specific antibodies. (A-F) Representative data from 3 independent experiments are shown. Histograms show densitometric data from 3 experiments. * p<0.05 control vs mutant cells.

Despite the inability of CD4^+^ T cells from mice and rats with mutations in the *Gimap5* gene to undergo robust proliferation in response to cognate antigens, as well as polyclonal stimulations such as cross-linking CD3 and CD28 or a combination of PMA and ionomycin, these cells were capable of differentiating into Th17 cells [31,36]. As differentiation into Th17 cells is associated with the activation of the mTORC1 complex [32–34], we assessed the mTORC1 pathway by analyzing the phosphorylation of its canonical substrates. Phosphorylation of 4EBP1 releases eIF4E to initiate cap-dependent translation [37]. Phosphorylation of S6K activates its kinase activity that results in the phosphorylation of ribosomal protein S6, a component of the 40S ribosome, which is one of the most characterized substrates of S6K [38]. Following cross-linking CD3/CD28 on the surface of CD4^+^ T cells, phosphorylation of the S6 peaked between 2 and 10 minutes in controls (Fig 1A). To our surprise, we observed that even in the absence of any stimulation, S6 and 4EBP1 were heavily phosphorylated in CD4^+^ T cells from *Gimap5* ^*sph/sph*^ mice (Fig 1A). It is possible that the polyclonality and plasticity of the T cell repertoire could have skewed the distribution of the T cell subsets that are present in the peripheral pool of *Gimap5* ^*sph/sph*^ mice. Therefore, we assessed the expression of pS6 and p4EBP1 in CD4^+^ T cells from OTII transgenic control and *Gimap5* ^*sph/sph*^ mice (OTII cells) following stimulation with OVA_323-339_ presented by irradiated APCs. OTII cells from control mice showed a gradual increase in pS6 protein over a period of 3 hours. However, similar to polyclonal T cells, OTII cells from *Gimap5* ^*sph/sph*^ mice showed elevated basal levels of expression of pS6 with minimal increase over time (Fig 1B). The increased phosphorylation of S6 was not unique to *Gimap5* ^*sph/sph*^ mice as loss of functional GIMAP5 resulted in increased phosphorylation of S6 and 4EBP1 in *Gimap5* ^*lyp/lyp*^ rats as well (Fig 1C). Thirty minutes after stimulation with anti-CD3/CD28, the band corresponding to pS6 was reduced in both control and *Gimap5* ^*sph/sph*^ T cells (Fig 1A). These observations suggest that, even if pS6 is constitutively activated, pathways leading to TCR-induced de-phosphorylation of pS6 are not affected by *Gimap5* deficiency.

As phosphorylation of 4EBP1 and S6 is mediated by signals from mTORC1, we addressed the role of mTORC1 in this phosphorylation. To this end, purified CD4^+^ T cells from control and *Gimap5* ^*sph/sph*^ mice were pre-treated with rapamycin, an inhibitor of mTORC1 activity. Pre-treatment with rapamycin inhibited the constitutive and TCR-induced phosphorylation of S6 and 4EBP1 in CD4^+^ T cells from control and *Gimap5* ^*sph/sph*^ mice (Fig 1D and 1E). Similarly, the constitutive phosphorylation of S6 and 4EBP4 observed in T cells from *Gimap5* ^*lyp/lyp*^ rats was also diminished by treatment with rapamycin (Fig 1F), indicating that the increased mTORC1 activity observed in CD4^+^ T cells, as a consequence of the loss of GIMAP5 functions, is comparable across species.

### Activation of AMPK is not altered by *Gimap5* mutation

**Fig 2:**
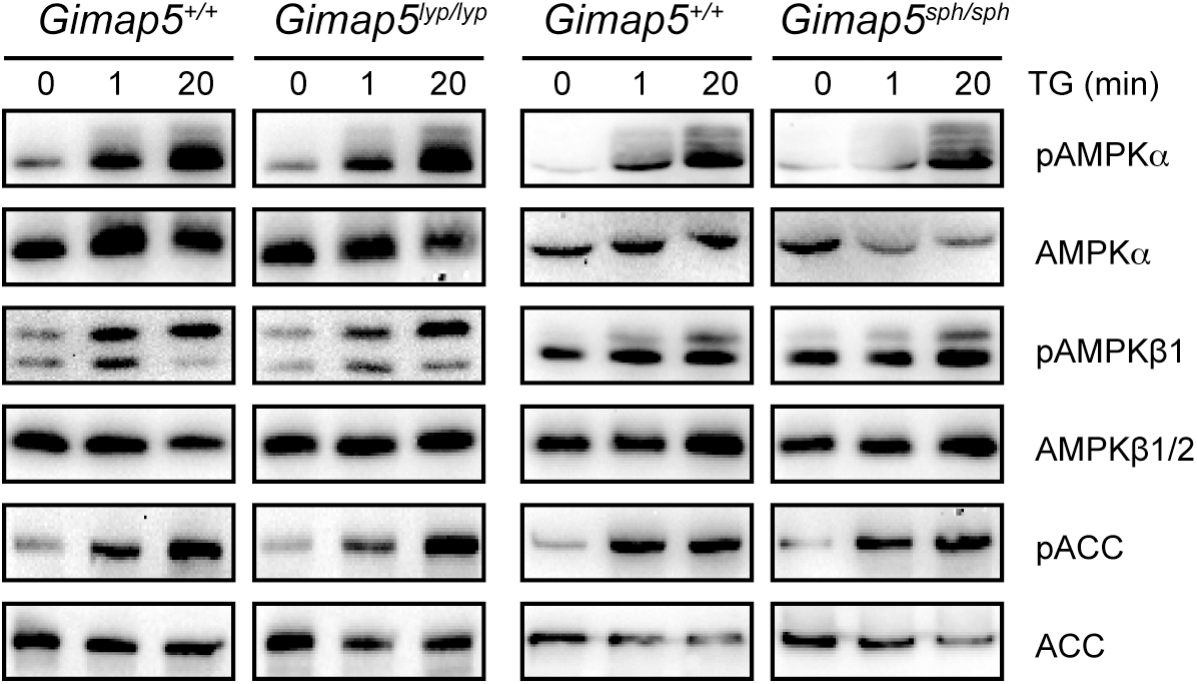
The inhibitory effect of GIMAP5 on mTORC1 activity is independent of AMPK. CD4^+^ T cells from control and *Gimap5* deficient rats or mice were left un-stimulated or stimulated with 1 μM TG for the indicated period. Cells were lysed and analyzed by western blot for the expression of the indicated proteins. Representative data from 3 independent experiments are shown.

T cell quiescence also requires the suppression of the mTORC1 complex by signals from the LKB1/AMPK pathway [20]. In quiescent T cells catabolic LKB1/AMPK pathway promotes mitochondrial oxidative phosphorylation (OXPHOS) and autophagy to meet the energy requirements. Cytosolic Ca^2+^ influx that follows TCR cross-linking can also transiently activate AMPK to increase the ATP production in anticipation of cell growth [39]. Therefore, we assessed the AMPK status in T cells at rest and following Ca^2+^ influx from intracellular stores. We have shown previously that Ca^2+^ release from the endoplasmic reticulum (ER), following TCR cross-linking, was not affected in T cells from *Gimap5* ^*lyp/lyp*^ rats [29]. To mimic the TCR-induced Ca^2+^ release from the ER we used thapsigargin (TG), a pharmacological inhibitor of the sarco/endoplasmic reticulum Ca^2+^-ATPase (SERCA) pump that is involved in the re-filling of the ER Ca^2+^ stores. As the analysis of AMPK signaling was not consistent after stimulation through CD3/CD28, we used TG to induce Ca^2+^ influx. As shown in Fig 2 we did not observe any difference between T cells from control and *Gimap5* deficient rats or mice in the basal or Ca^2+^-induced phosphorylation of AMPK following the addition of TG. Furthermore, the pattern of phosphorylation of ACC, the downstream substrate of AMPK was also comparable between the control and *Gimap5* mutants in both the rats and in mice. These results suggest that *Gimap5* does not influence the activation of the AMPK pathway in T cells.

**Fig 3:**
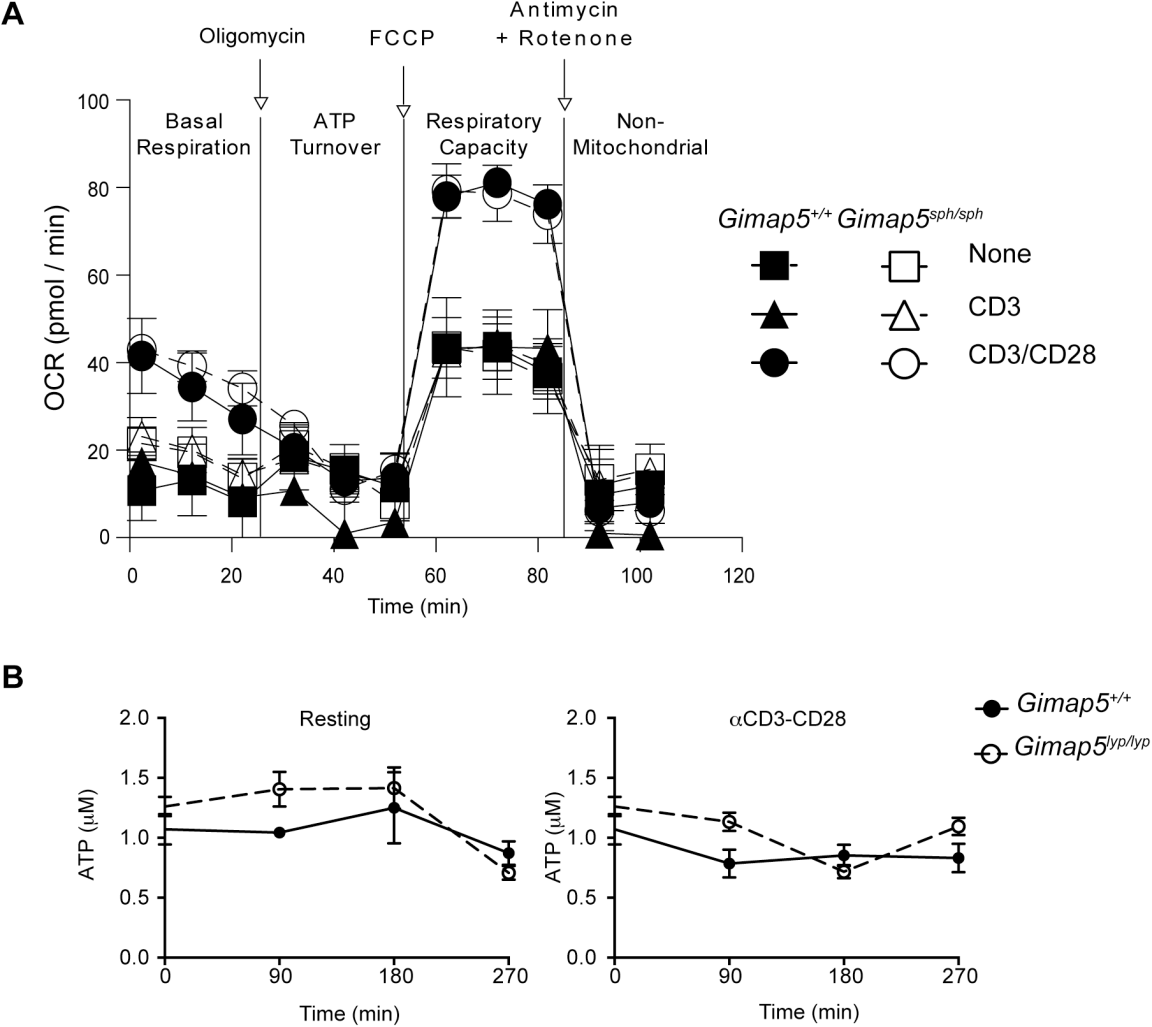
Mitochondrial respiration is normal in *Gimap5*-deficient CD4^+^ T cells. (A) CD4^+^ T cells from control and *Gimap5* deficient mice were left un-stimulated or stimulated with 5 μg/mL anti-CD3 or anti-CD3/CD28 for overnight. Metabolic flux was determined using Seahorse Biosciences XF96 Analyzer. Measurement of OCR is shown under basal conditions and following sequential addition of oligomycin, FCCP, and rotenone + antimycin A. Representative data from 3 independent experiments carried out in triplicate are shown. (B) CD4^+^ T cells from control and *Gimap5* ^*lyp/lyp*^ rats were unstimulated or stimulated with CD3/CD28 for indicated duration. ATP levels were measured using the bioluminescence assay. Pooled data from 3 independent experiments are shown.

Energy requirements of quiescent cells are mostly met from catabolic processes involving mitochondrial oxidative respiration [40]. As *Gimap5* mutation results in the loss of mitochondrial membrane potential over time and reduced ability of mitochondria to buffer cytosolic Ca^2+^ [30], we assessed whether CD4^+^ T cells from *Gimap5* ^*sph/sph*^ mice showed alterations in OXPHOS. We assessed mitochondrial respiration using an extracellular flux analyzer. Here, alterations in OXPHOS are reflected in the amount of oxygen consumed by the mitochondria, expressed as oxygen consumption rate (OCR). Again, we did not observe any difference in the OCR between T cells from control and *Gimap5* ^*sph/sph*^ mice (Fig 3A). Similarly, mutation in *Gimap5* did not affect the energy production in resting T cells or following cross-linking of CD3/CD28 as measured by the total ATP content in these cells (Fig 3B). Taken together, these observations suggest that *Gimap5* does not directly influence the energy content in T cells.

### PP2A phosphatase activity is not altered in *Gimap5*-deficient T cells

**Fig 4:**
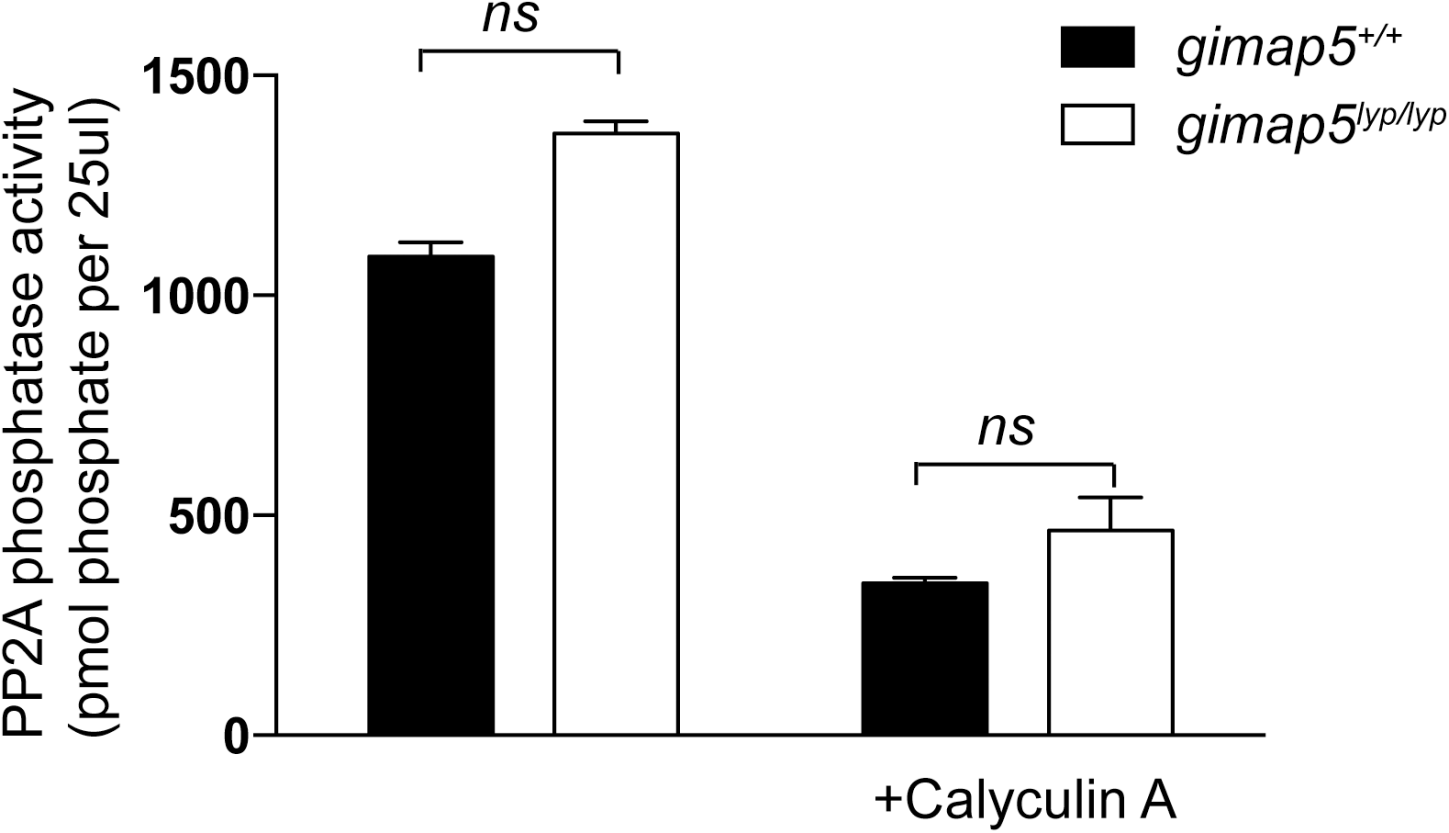
GIMAP5 inhibits mTORC1 signaling in a PP2A-independent manner. CD4^+^ T cells from control and *Gimap5* ^*lyp/lyp*^ rats were unstimulated or pretreated with Calyculin A for 30 min. Then the cells were lysed and the lysates was immunoprecipitated with anti-PP2A antibody. The immunoprecipitates were washed with TBS buffer followed by phosphatase reaction buffer. PP2A activity was assayed according to the manufacturer’s instructions. Data has been pooled from 2 independent experiments, carried out in duplicate.

The constitutive phosphorylation of S6 and 4BP1 in T cells from *Gimap5* mutant mice and rats could also arise from suboptimal phosphatase activity. PP2A, a serine/threonine protein phosphatase, is the major phosphatase responsible for the de-phosphorylation of S6 and 4EBP1 [41]. An earlier study had shown that apoptosis induced by okadaic acid (OA), an inhibitor of the PP2A could be prevented by transfection with human *GIMAP5* (previously called *IAN5*) gene [42]. As a corollary, we have also observed that *Gimap5* ^*lyp/lyp*^ T cells lacking functional *Gimap5* showed increased sensitivity to OA-induced apoptosis (S1 Fig). We confirmed the role of PP2A in dephosphorylating S6 and 4EBP1 by treating the cells with calyculin A, an inhibitor of PP2A [43]. As expected, treatment with calyculin A resulted in an increased phosphorylation of S6, 4EBP1 and AKT (S2 Fig). To determine whether the constitutive phosphorylation of S6 and 4EBP1 could be the result of decreased PP2A activity, we measured the activity of PP2A in the lysates of CD4^+^ T cells from *Gimap5* ^*lyp/lyp*^ rats. Under steady state conditions, the activity of PP2A was comparable in CD4^+^ T cells from *Gimap5* deficient and control rats (Fig 4). These observations suggest that the persistence of the phosphorylation of S6 and 4EBP1 cannot be attributed to decreased phosphatase activity, per se.

### *Gimap5* deficiency results in constitutively active AKT

**Fig 5:**
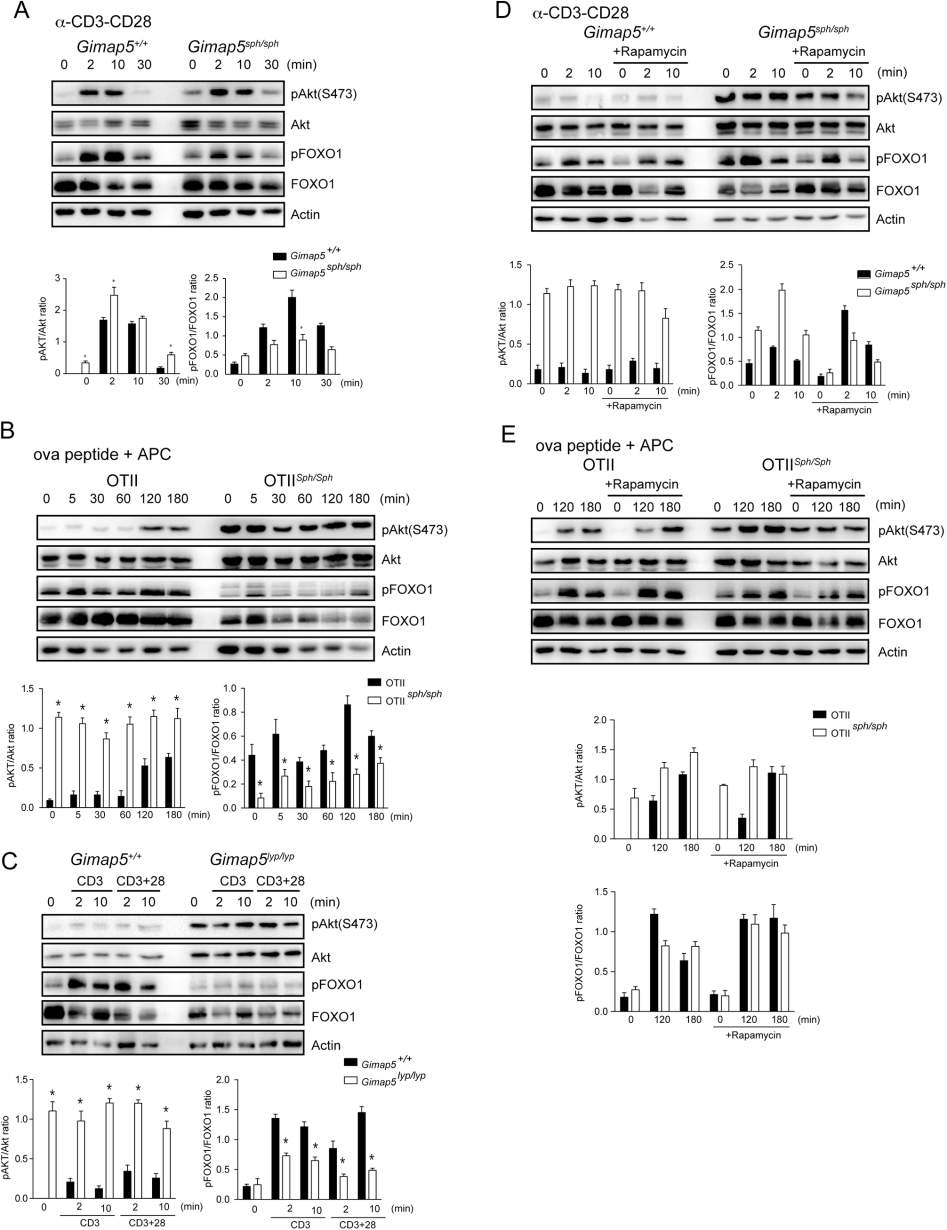
*Gimap5* mutation results in constitutive activation of AKT. (A and C) CD4^+^ T cells from control and *Gimap5* deficient mice (A) and rats (C) were stimulated with 5 μg/mL anti-CD3 or anti-CD3/CD28 for the indicated duration. Lysates were analyzed by Western blot with the indicated antibodies. (B and E) CD4^+^ T cells from the OT-II TCR-transgenic control and *Gimap5* ^*sph/sph*^ mice were simulated with OVA peptide presented by APC in the absence (B) or presence of rapamycin (E). Cell lysates were probed with specific antibodies. (D) CD4^+^ T cells from control and *Gimap5* deficient mice were treated with 200 nM rapamycin for 30min followed by TCR crosslinking at different time points. Cell lysates were analyzed by Western blot using indicated antibodies. (A-E) Representative data from 3 independent experiments are shown. Histograms show densitometric data from 3 experiments. * p<0.05 control vs mutant cells.

**Fig 6:**
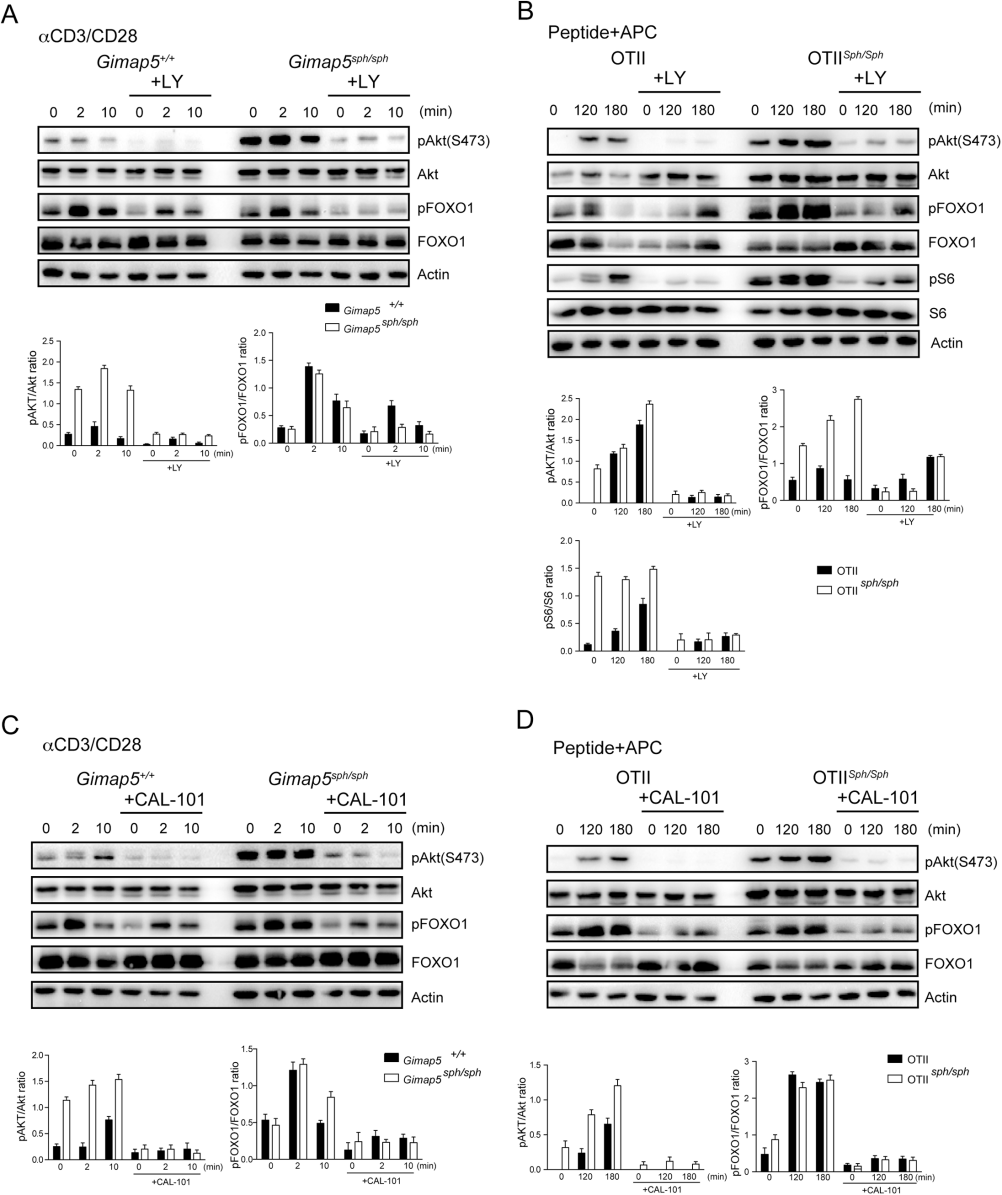
Aberrant activation of PI3K contributes to hyper-activated AKT in *Gimap5* deficient CD4^+^ T cells. (A and C) CD4^+^ T cells from control and *Gimap5* ^*sph/sph*^ mice were treated with 10 μM LY294002 (A) or 1 μM CAL–101 (C) for 30 min followed by TCR stimulation. The lysates were analyzed with indicated antibodies. (B and D) CD4^+^ T cells from the OT-II TCR-transgenic *Gimap5* ^*sph/sph*^ and control mice were stimulated with antigen in the presence of LY294002 (B) or CAL–101 (D). Cell lysates were probed with specific antibodies. (A-D) Representative data from 3 independent experiments are shown. Histograms show densitometric data from 3 experiments. * p<0.05 control vs mutant cells.

Cross-linking of TCR/CD3 complex results in the recruitment of PI3K to the CD3 complex, to generate phosphatidylinositol (3,4,5)-triphosphate (PIP3) at the plasma membrane [44]. Phosphoinositide-dependent kinase–1 (PDK1) and AKT are recruited by PIP3 through their pleckstrin homology (PH) domains leading to the phosphorylation of AKT at Thr308, followed by the phosphorylation of Ser473 by the mTORC2 complex [45]. Activated AKT phosphorylates the TSC1/TSC2 complex, resulting in the inhibition of its GTPase-activating protein (GAP) activity and releasing the RHEB GTPase to activate mTORC1 [46]. We assessed whether the increased phosphorylation of AKT following cross-linking of TCR/CD3 complex and CD28 could explain the increased mTOR activity. In CD4^+^ T cells from control mice, we observed that the pAKT peaked between 2 and 10 minutes after CD3/CD28 cross-linking, and returned to the basal level by 30 minutes (Fig 5A). In contrast, phosphorylation of AKT was detected even in the absence of stimulation in CD4^+^ T cells from *Gimap5* ^*sph/sph*^ mice (Fig 5A). Crosslinking CD3/CD28 also increased the pAKT that returned to basal level within 30 minutes in *Gimap5* ^*sph/sph*^ CD4^+^ T cells. But it was still higher than that observed in the control cells. Following activation of control OTII cells with peptide/APC, phosphorylation of AKT was evident at 5 minutes and was strong even at 180 minutes (Fig 5B). However, in *Gimap5* mutant OTII cells, pAKT was detected even in the absence of stimulation and showed minimal increase over time (Fig 5B). Increased basal AKT phosphorylation was also observed in CD4^+^ T cells from *Gimap5* ^*lyp/lyp*^ rats (Fig 5C). These results strongly indicated that loss of functional GIMAP5 protein results in elevated basal phosphorylation of AKT in T cells. A previous report has shown that the expression of FOXO1, the down stream target of AKT, is reduced in CD4^+^ T cells from 4-week old *Gimap5* ^*sph/sph*^ mice [31]. We observed that the phosphorylation of FOXO1 is comparable or lower in *Gimap5* mutant CD4^+^ T cells even in the presence of hyperactivated Akt (Figs 5 and 6). Furthermore, to confirm that the activation of AKT was not an artifact, we determined the phosphorylation status of AKT and FOXO1 in the cells where mTOR was inhibited. While phosphorylation downstream of mTORC1 was inhibited following pre-treatment with rapamycin (Fig 1D, 1E and 1F), phosphorylation of AKT and FOXO1 remained unchanged (Fig 5D and 5E). These results indicate that the constitutive activation of AKT likely underlies the activation of the mTORC1 pathway in T cells lacking functional GIMAP5 protein.

### Constitutively activated PI3K is responsible for the increased pAKT in GIMAP5 deficient T cells

Engagement of the TCR or IL-7R activates PI3K to generate PIP3 at the plasma membrane which results in the recruitment and activation of AKT [47]. To determine whether the phosphorylation of AKT was secondary to the activated state of PI3K, we pretreated the cells with different inhibitors of PI3K. LY294002 (LY) and CAL–101 markedly diminished the intensity of the pAKT in CD4^+^ T cells from control and *Gimap5* ^*sph/sph*^ mice and *Gimap5* ^*lyp/lyp*^ rats (Fig 6 and S3 Fig). Phosphorylation of AKT following activation of OTII cells with ova peptide was also completely prevented by CAL–101 (Fig 6D). More importantly, inhibition of PI3K greatly diminished the amount of constitutive pAKT, indicating that aberrant activation of PI3K may be the underlying cause for the activation of the AKT/mTOR pathway. In addition, the level of pFOXO1, an mTORC1-independent substrate of pAKT was also reduced following treatment with PI3K inhibitors (Fig 6). The pattern of inhibition between LY and CAL–101 was comparable in control and mutant cells (Fig 6A versus 6C and Fig 6B versus 6D). While LY is pan PI3K inhibitor, CAL–101 is specific to PI3Kdelta isoform that is active in lymphocytes [44]. Thus, it is reasonable to conclude that the constitutive phosphorylation of AKT is essentially mediated by PI3K delta in the T cells from *Gimap5* mutant mice.

## Discussion

Long-term survival of T lymphocytes in a quiescent state is essential to maintain their cell numbers in the peripheral circulation and in secondary lymphoid organs. Even though cytokines, T cell antigen receptor and various signaling pathways are implicated in the maintenance of T cell survival, the processes by which these signaling pathways are integrated are not yet well understood. In BB-DP rats, T lymphopenia, that arises due to the recessive mutation in the *lyp* allele, promotes abortive homeostatic expansion of T cells resulting in autoimmune diabetes [6] (reviewed in [7]). As a consequence of this mutation, single positive thymocytes and mature T lymphocytes undergo spontaneous apoptosis [48,49]. Twenty years after its discovery, the mutation was mapped to the *Gimap5* locus (formerly *Ian5*) leading to the discovery of a new family of proteins [3,4]. A GTPase domain in the N-terminus that can potentially hydrolyze GTP characterizes GIMAP proteins. Some of these possess a C-terminal hydrophobic trans-membrane domain, which targets GIMAP5 to the endo-lysosomal compartment [50]. While in rats the functional consequence of *Gimap5* mutation is restricted to the T cell lineage, in mice it extends to other hematopoietic cells and prevents the survival of hematopoietic stem cells [8,9].

In *Gimap5* ^*lyp/lyp*^ rats the frame-shift mutation truncates the coding sequence at amino acid 109 such that the putative polypeptide product lacks both a functional GTPase domain and the C-terminal membrane anchor. [3,4]. In *Gimap5* ^*sph/sph*^ mice a point mutation disrupts the functional GTPase domain. While the mRNA is expressed, the protein is not detected, probably due to its lack of stability [9]. These observations suggest that the GTPase domain of GIMAP5 protein may be functional. Studies with GIMAP2 indicate that the GTPase domain in GIMAP2, 7 and 5 can bind GTP and can potentially hydrolyze GTP when they form dimers [51]. GIMAP5 has comparable functions, at least in T cells, in both rats and mice as observed in this study where we have carried out most of the experiments in both *Gimap5* mutant rats and mice.

Most of the GIMAP proteins are expressed in the cells of the hematopoietic system [52]. However, the mechanisms by which GIMAP proteins promote the survival of T and B lymphocytes are far from well understood. In mice, GIMAP5 has been reported to interact with the members of the Bcl–2 family [2,10]. However, we did not observe co-localization of Bcl–2 with rat GIMAP5 in primary rat T lymphocytes or following overexpression [35]. Our observations rather suggest that GIMAP5 may be involved in the integration of the survival signals generated through the TCR by regulating the calcium homeostasis in T lymphocytes [29]. In fact the TCR-induced release of Ca^2+^ from the ER stores is normal in GIMAP5 deficient T cells suggesting that certain aspects of the proximal TCR signaling machinery is intact in these cells [29]. The reduced Ca^2+^ influx may possibly be attributable to the inability of the mitochondria to move on microtubules to buffer the cytosolic Ca^2+^ in the absence of functional GIMAP5 protein [30]. A similar function has been recently reported for GIMAP4 that interacts with actin and tubulin for cytokine secretion in CD4^+^ T cells [53]. These observations collectively raise the possibility that the survival defect in these *Gimap5* mutant T cells can also, in part, be due to defects in vesicular traffic.

Despite the lymphopenia and the defective proliferative response following TCR stimulation, *Gimap5*-deficient T cells do differentiate into Th17 cells and contribute to tissue pathologies [9,36,54]. T cell-specific deletion of regulatory associated protein of mTOR (RAPTOR), the mTOR binding partner that also binds p70S6k and 4E-BP1, prevents the down-regulation of Gfi1, a negative regulator of Th17 differentiation [55]. Our observations show that this skewing towards Th17 differentiation in *Gimap5*-deficient T can be attributed to the increased mTORC1 signaling (Fig 1). As a consequence, *Gimap5*-deficient T cells show a decrease in the Treg population, which could also aggravate the observed pathologies [9,56]. However, it is not clear if the observed phenotypes are secondary to lymphopenia. Competitive reconstitution leads to the loss of the mutant T cells over time in rats, making it difficult to address the role of lymphopenia [49]. However, reconstitution with *Gimap5*-sufficient cells greatly reduced the development of autoimmune diabetes in the rat model and the intestinal lesions in the mouse model [7,9], suggesting that the manifestation of the pathologies associated with the *Gimap5* mutation are secondary to the lymphopenia-induced activation.

Quiescence in T cells also requires the repression of mTORC1 pathway by the LKB1/AMPK axis that promotes catabolic pathways involving mitochondrial OXPHOS [20]. The activation of the mTORC1 pathway is not due to defects in AMPK activation as seen from the equivalent activation of AMPK and the mitochondrial OXPHOS in *Gimap5* deficient T cells (Figs 2 and 3). Rather, *Gimap5*-deficient T cells show spontaneous activation of AKT, which then activates the mTORC1 pathway through the suppression of the TSC complex (Fig 5). The constitutive activation of AKT appears to be specific to T lymphocytes as it is not observed in B cells, even though their survival is also affected in the absence of GIMAP5 [9]. Thus, activation of the mTORC1 pathway can be attributed to the spontaneous activation of the PI3K/AKT axis.

T cells require two signals to sustain T cell activation, cytokine production and cell proliferation [57]. The primary signal through the TCR needs to be accompanied by a second signal through the co-stimulatory molecules. Signals through the TCR complex alone, in the absence of co-stimulation, result in the development of anergic T cells that cannot easily respond to robust re-stimulation through the TCR [58]. Recently, two groups have reported that spontaneous gain of function mutations in PI3K resulted in profound immunodeficiency, as these T cells were unable to mount an efficient immune response to cross-linked anti-CD3/CD28 stimulation [59,60]. Similarly, here we show that as a consequence of the mutation in *Gimap5*, the PI3K/AKT axis is constitutively activated in the absence of spontaneous activation of TCR-mediated proximal signaling pathways. It has been shown that mutations in PI3K can alter the signaling threshold during T cell development [61]. However analysis of the phenotype of thymocytes in *Gimap5* mutant rats and mice suggest that GIMAP5 is required for T cell survival during later stages of T cell development in the thymus and in the periphery. We hope that the dissection of the signaling pathways in GIMAP5-deficient cells will eventually contribute to the understanding of the mechanisms through which GIMAP5 promotes the survival of lymphocytes.

## Supporting Information

S1 Fig *Gimap5* ^*lyp/lyp*^ T cells are more sensitive to OA induced apoptosis. Total lymphocytes from control and *Gimap5* ^*lyp/lyp*^ rat were cultured with or without 200 nm OA for 8 h. The apoptosis in gated CD4^+^ lymphocytes was analyzed by annexin V staining using flow cytometry. The number of cells recovered from cultures of lymphocytes *Gimap5* ^*lyp/lyp*^ rats diminishes with time. As a consequence only few CD4^+^ cells can be acquired in the live gate. Representative data from 3 independent experiments are shown.
(TIF)

S2 Fig PP2A inhibitor markedly increased the basal level of phosphorylation of PP2A substrates. CD4^+^ T cells from wild type mice were treated with PP2A specific inhibitor calyculin A for 1 h. The phosphorylation of S6, 4EBP1 and AKT was analyzed by Western blotting. Representative data from 2 independent experiments are shown.
(TIF)

S3 Fig Inhibition of PI3K diminished the phosphorylation of AKT in both control and *Gimap5* deficient CD4^+^ T cells. CD4^+^ T cells from control and *Gimap5* ^*lyp/lyp*^ rats were treated with 1 μM CAL–101 for 30 min followed by TCR stimulation. The lysates were analyzed with indicated antibodies. Representative data from 3 independent experiments are shown.
(TIF)

## Abbreviations

GIMAP: GTPase of the immune associated nucleotide binding protein
mTOR: mammalian target of rapamycin
PP2A: protein phosphatase 2A
ACC: acetyl CoA carboxylase
AMPK: AMP-activated protein kinase
PI3K: phosphoinositide 3-kinase
BB-DP: diabetes prone Bio Breeding
TCR: T cell receptor
IL-7R: interleukin–7 receptor
JAK/STAT: Janus kinase/signal transducers and activators of transcription
LKB1: liver kinase B1
mTORC1: mTOR complex 1
S6K: S6 kinase
TSC1/2: tuberous sclerosis complex
RHEB: RAS homologue enriched in brain
eIF-4E: suppression of eukaryotic initiation factor 4E
4EBP1: eIF-4E binding protein 1
Ca^2+^: calcium
FOXO: forkhead box O
FCCP: fluoro-carbonyl cyanide phenylhydrazone
FBS: fetal bovine serum
TG: thapsigargin
OA: okadaic acid
ECL: enhanced chemiluminescence
APC: antigen-presenting cells
GAPDH: glyceraldehyde-3-phosphate dehydrogenase
OCR: oxygen consumption rates
OXPHOS: oxidative phosphorylation
ER: endoplasmic reticulum
SERCA: sarco/endoplasmic reticulum Ca^2+^-ATPase
LAT: linker for activation of T cells
PIP3: phosphatidylinositol (3,4,5)-trisphosphate
PDK1: phosphoinositide-dependent kinase–1
GAP: GTPase-activating protein
IP3: inositol tri-phosphate
RAPTOR: regulatory associated protein of mTOR
