# Supplementary figures and images for: GIMAP5 Deficiency Is Associated with Increased AKT Activity in T Lymphocytes
Source: PLoS One. 2015 Oct 6;10(10):e0139019. doi: 10.1371/journal.pone.0139019 (PMC4595448; doi:10.1371/journal.pone.0139019)

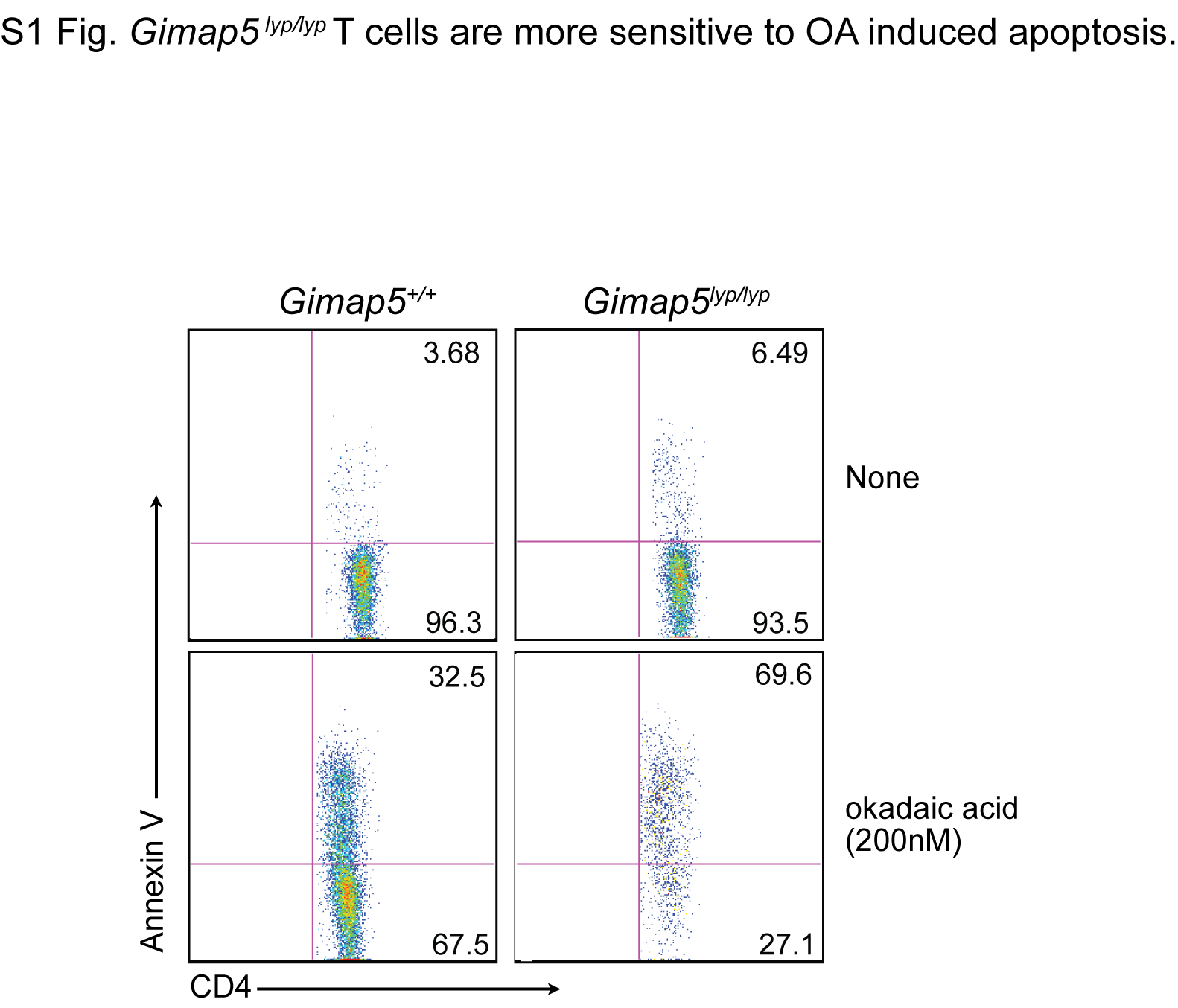

Supplement: S1 Fig — Total lymphocytes from control and Gimap5 lyp/lyp rat were cultured with or without 200 nm OA for 8 h. The apoptosis in gated CD4+ lymphocytes was analyzed by annexin V staining using flow cytometry. The number of cells recovered from cultures of lymphocytes Gimap5 lyp/lyp rats diminishes with time. As a consequence only few CD4+ cells can be acquired in the live gate. Representative data from 3 independent experiments are shown. (TIF) [file pone.0139019.s001.tif]

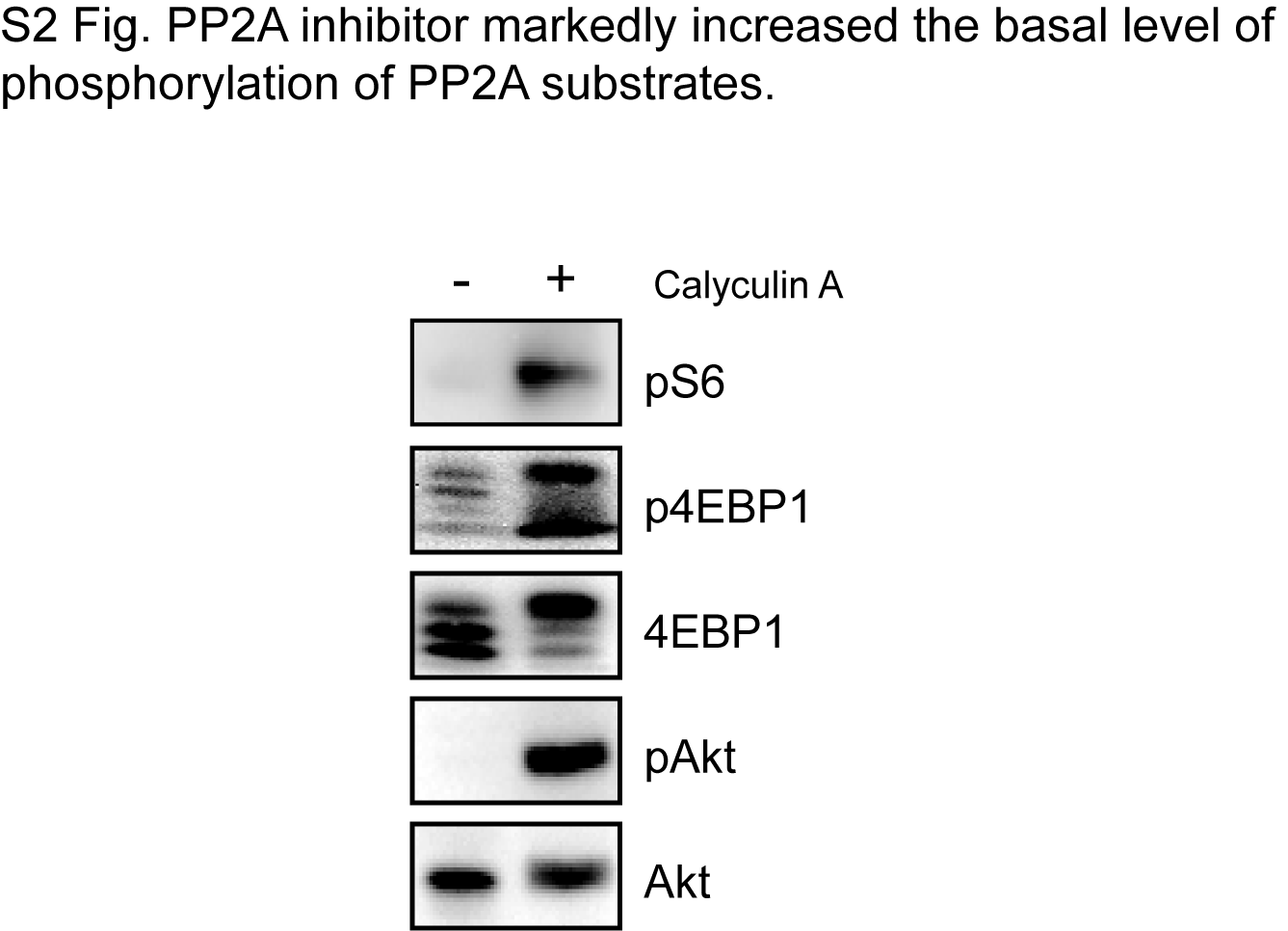

Supplement: S2 Fig — CD4+ T cells from wild type mice were treated with PP2A specific inhibitor calyculin A for 1 h. The phosphorylation of S6, 4EBP1 and AKT was analyzed by Western blotting. Representative data from 2 independent experiments are shown. (TIF) [file pone.0139019.s002.tif]

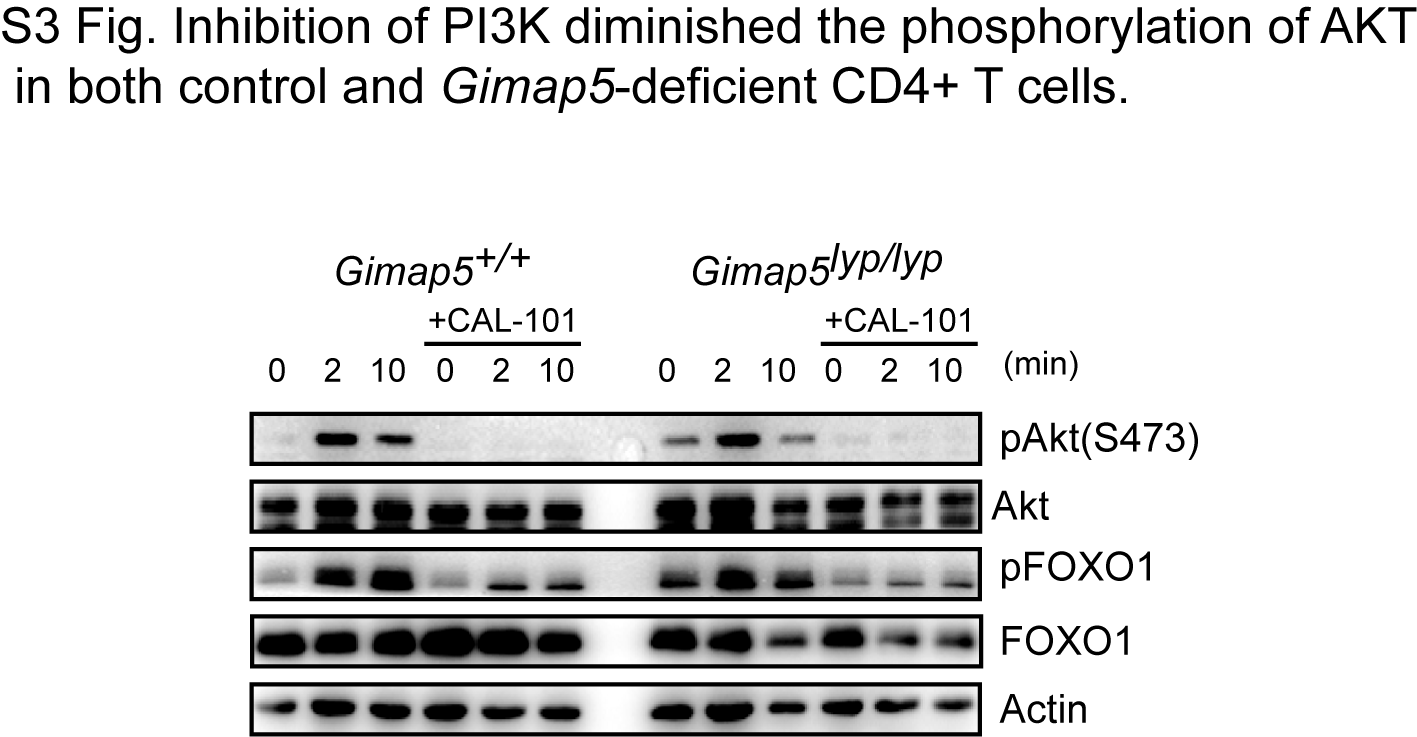

Supplement: S3 Fig — CD4+ T cells from control and Gimap5 lyp/lyp rats were treated with 1 μM CAL–101 for 30 min followed by TCR stimulation. The lysates were analyzed with indicated antibodies. Representative data from 3 independent experiments are shown. (TIF) [file pone.0139019.s003.tif]
